# Intraoperative prevention of Surgical Site Infections as experienced by operating room nurses

**DOI:** 10.1080/17482631.2019.1632109

**Published:** 2019-07-01

**Authors:** Maria Qvistgaard, Jenny Lovebo, Sofia Almerud-Österberg

**Affiliations:** Department of Health and Caring sciences, Linneus University, Växjö, Sweden

**Keywords:** Intraoperative care, Operating room, Prevention, Surgical Site Infection, Surgical team

## Abstract

**Aim**: This study examines how OR nurses experience intraoperative prevention of SSIs.

**Introduction**: Infections related to surgical procedures create both great patient suffering and high costs for society. Therefore, prevention of Surgical Site Infections (SSIs) should be a high priority for all surgical settings. All details of intraoperative care need to be investigated and evaluated to ensure best practices are evidence-based.

**Methods**: This study uses the Reflective Lifeworld Research (RLR) approach, which is grounded in phenomenology. Participants were OR nurses with at least one year of clinical experience. In total, 15 participants from seven hospitals made contact and were included in this interview study.

**Results**: Prevention of SSIs takes both head and hand. It requires long-term, continuous, and systematic work in several parallel processes, both intellectually and organisationally. The hierarchical tradition of the operating room is often ambiguous, shielded by its safe structures but still restricted by traditional patterns. Confident relations and resolute communication within the team generate favorable conditions for preventing SSIs.

**Conclusions**: By setting up mutual platforms and forums for quality development, increasing legitimacy for OR nurses and establishing fixed teams, prevention of SSIs will continue to improve, ensuring the patients’ safety during intraoperative care.

## 1. Introduction

Infections related to surgical procedures create both great patient suffering and high costs for society. Therefore, prevention of Surgical Site Infections (SSIs) should be a high priority for all surgical settings (Andersson, Bergh, Karlsson, & Nilsson, 2010). Although the prevalence of SSIs is much higher in low- and middle-income countries compared to high-income countries, SSIs are still a common cause of infection in high-income countries (Allegranzi et al., 2016). Clearly, better SSIs prevention is needed. Preventing SSI requires a multifactorial approach as the increasing development of antibiotic resistance makes it essential that the Operating Room (OR) is as clean as possible.

SSIs usually occur within 30 days of surgery, but in some cases (e.g., after orthopedic joint surgery) SSIs manifest much later. The major risk of infection is microorganisms breaching surgical incisions (Harrington, 2014). The development of SSIs depends on virulence, bacterial load, and the patient’s ability to resist infections (Mockford & O’Grady, 2017). The lack of knowledge about the risk of resistance has lead to the rapid increase in antibiotic resistance (Laxminarayan et al., 2013). The World Bank Group estimates substantial losses in the global economy related to this issue. The damage inflicted by antibiotic resistance will continue for a long time and people in low-income countries will suffer the most (Adeyi et al., 2017).

The bacterial load in the surgical environment can be controlled by implementing proven guidelines that reduce the risk of SSIs during intraoperative care. Actions such as all staff complying with hygienic practices and accurate cleaning of the OR between operations are examples of measures that can reduce bacterial load, an outcome that will inevitably reduce SSIs (Liu et al., 2018). However, clinical practice compliance is difficult to maintain. To address this problem, more evidence about the effects of SSIs prevention is needed (Leaper, Tanner, Kiernan, Assadian, & Edmiston, 2015).

As most SSIs are avoidable (Mellin-Olsen, McDougall, & Cheng, 2017), it is likely that continued research on optimal OR conditions will benefit patients. All details of intraoperative care need to be investigated and evaluated to ensure best practices are evidence-based. That is, evidence of the effect of several care bundles related to SSIs (e.g., preoperative skin disinfection with chlorhexidine) is insufficient (Webster & Osborne, 2015). Global guidelines for safe surgery emphasis that preventable care bundles are complex and demand multidisciplinary collaboration (Lambrecht, 2008). Existing evidence suggests that communication lapses in the OR are common and these lapses affect patient outcome, sometimes resulting in SSIs (Pugel, Simianu, Flum, & Patchen Dellinger, 2015). This less than effective communication has been attributed to nurses experiencing lower quality teamwork than what surgeons experience (Carney, West, Neily, Mills, & Bagian, 2010).

Prevention of SSI is a balance between harm, cost, and patient values (Allegranzi et al., 2016). Surgical patients, who meet many health care professionals on their perioperative journey, rely on OR nurses to provide effective care as well as to ensure the prevention of SSIs. That is, OR nurses are responsible for implementing hygiene and aseptic principles in the OR to prevent and limit the spread of infections. In addition, OR nurses are responsible for perioperative care together with other specialists in the team (Nordström et al., 2019).

To date, qualitative research has not described the intraoperative prevention of SSI from the perspective of OR nurses. A Scottish study, however, shows that non-technical skills such as situational awareness, good communication, and coping with stress are essential skills required for OR nurses to effectively perform safe care practices in the intraoperative phase (i.e., during the surgical procedure) (Mitchell et al., 2011). To ensure the sustained presence of OR nurses’ competence during perioperative care, there is a need to capitalise on their important contributions to the prevention of SSIs (Gillespie & Pearson, 2013). Hence, this study examines how OR nurses experience intraoperative prevention of SSIs.

## 2. Material and methods

This study uses the Reflective Lifeworld Research (RLR) approach, which is grounded in phenomenology. From a phenomenological perspective, the researcher tries to be as open as possible to both generalities and particularities of the phenomenon and its meaning. Methodological principles of RLR are openness, bridling, and reflection, principles that attempt to grasp the essence of a phenomenon and its variations (Dahlberg, Dahlberg, & Nyström, 2008). The phenomenon investigated in this study is intraoperative prevention of SSIs as it is experienced by OR nurses.

### 2.1. Participants and setting

Participants were OR nurses with at least one year of clinical experience. In Sweden, studying to become an OR nurse requires three years of nursing education and a one-year master’s degree. The participants were employed as OR nurses in southern Sweden. At each hospital, a recruitment advertisement was posted in a frequently used area (e.g., a break room). OR nurses interested in more information about the study or who wanted to participate were encouraged to contact the first author. These potential participants were not pressured to take part in the study. In total, 15 participants from seven hospitals made contact and were included in the study. Clinical experience as an OR nurse varied among the participants (2–34 years). In this population, women are in the majority, which is reflected in the sample. The informants had varied experience in general surgery, orthopedics, and gynecology and were employed in variety of surgical settings, ranging from small to large.

### 2.2. Data collection

One separate interview with each participant was performed between spring and autumn 2016. The phenomenon-oriented open interviews lasted between 38 and 75 minutes. All but two interviews, which were conducted at the participants’ homes, were conducted at the participant’s workplace during work hours, with permission from the department heads. The interviews were conducted and audio recorded by the first author. The participants were released from work-related tasks so the interviews could take place without interruptions. Shortly after the interviews, the first author transcribed the interviews verbatim. After the interview, the participants were encouraged to make contact if they wanted to change or add to their interview although none chose to do so. All interviews had the same starting question: “What does SSI prevention mean to you in your everyday work as an OR nurse?”. On some occasions, there was a need to ask clarifying questions such as “Can you please take me through a day in your profession and describe how you work with prevention of SSIs?”. To deepen some descriptions, the interviewer continued with follow-up questions such as “How do you mean?” and “Can you give me an example?”

### 2.3. Data analysis

The analysis started during the transcription of the interviews and continued with repeated readings of each interview to become familiar with the data. Parts of the text that obviously and clearly related to the phenomenon were identified and marked as meaning units. The first author and co-authors searched the transcriptions for meaning units. To bridle the pre-understanding of the phenomenon during the analysis, all authors discussed the meanings and understanding of the meaning units. A diverse professional background within the group of authors prevented preunderstanding to unreflexively influence the analysis. Next, the authors searched for patterns (i.e., similarities and differences in the meanings) to create clusters of meaning. From the clusters of meaning, a core meaning became clear—this core is the phenomenon’s essence. The essence is what makes a phenomenon the phenomenon while its constituents are the particulars of the phenomenon’s totality (Dahlberg et al., 2008). No software programs were used for data analysis. In the following, the team refers to a surgical team with several professions including surgeons, OR nurses, and nurse anesthetists.

### 2.4. Ethical considerations

This study was approved by the Ethical Regional Committee in Linkoping, Sweden (Dnr 2016/73–31). Ethical considerations were performed in accordance with the Declaration of Helsinki (World Medical Association, 2013). All participants received both written and verbal information about the study. Before the interviews took place, the participants signed a written consent and were ensured confidentiality. The heads of the departments in all the OR units were informed about the study and agreed to let their nurses be interviewed during work hours at work or another place of the participants’ choosing.

## 3. Results

The results are presented as the essence of the studied phenomenon: intraoperative prevention of SSIs as experienced by OR nurses. The essence is further described in the constituents, which further elucidate variations of the phenomenon.

Intraoperative prevention of SSIs is a struggle against an invisible threat. The powerful threat that microorganisms constitute in the surgical environment is a significant risk to patients and is the foundation understanding for developing prevention strategies and techniques. Prevention of SSIs takes both head and hand. It requires long-term, continuous, and systematic work in several parallel processes, both intellectually and organisationally. Vigilance, strict adherence to details, inspection, and surveillance of sterile fields are essential aspects in preventing SSIs. Health care professionals are charged with using these prevention skills and techniques to shelter the patient during surgery. That is, preventing SSIs requires the professional skill of balancing well-judged decisions related to patient safety.

Although both theoretical and clinical knowledge of preventive principals within the operating room conveys confidence among team members, further competencies such as experience and courage are needed to ensure prevention. The hierarchical tradition of the operating room is often ambiguous, shielded by its safe structures but still restricted by traditional patterns. The balance between legitimacy and hierarchical tradition is fragile. Prevention of SSIs depends on an open and honest atmosphere within the team, a team that allows different professionals to contribute with their unique competencies. Awareness of risks related to SSIs is carried out by individuals who have confidence in each other and dare to confront human shortcomings. Confident relations and resolute communication within the team generate favorable conditions for preventing SSIs. A legitimate leader, irrespective of level in the organization, has the ability to balance risks and provide safety within a complex milieu such as an OR.

The following constituents describe the variations and nuances of the phenomenon: (1) a struggle against an invisible threat; (2) balancing risks and safety; and (3) legitimacy improves stability in a complex milieu.

### 3.1. A struggle against an invisible threat

OR personnel are committed to the fundamental tasks of their profession, which includes the promise that their patients will be “watched over” during the surgical procedure. An optimal surgical environment requires a sterile field—i.e., the removal of potential risks of contamination. The invisible threat of microorganisms can be made visible to others if OR nurses use their profound knowledge to explain the connection between bacterial load and the risk of SSIs. Prevention of SSIs in the OR requires rigorous attention to keeping microorganisms out of the surgical wound. That is, the team members should adhere to the guidelines regarding OR hygiene:

Everybody has their own responsibility inside the OR, but my job is to tell you when you are too close, when you have to change gloves, or when you need to adjust your surgical gown. You are trying to have an overview of everything that happens inside the OR and simultaneously keep the focus on what is going on during the surgical procedure.

Despite prevention, SSIs sometime arise after a surgical procedure although the SSIs are seldom reported to the team as a unit. The absence of structured feedback makes it difficult when the profession seeks arguments for strengthening routines related to preventing SSIs. Therefore, measures intended to combat this invisible threat are difficult to evaluate and analyse. This lack of evidence for the effectiveness of routines results in insecurity and doubt connected to SSIs prevention measures. In addition, this lack of evidence also brings hesitance towards fundamental grounds in the profession. However, the struggle against an invisible threat relies on a strong will to organise a protected zone where the surgical procedure can proceed safely: “Sterility is the alpha and omega to me; here is where my occupational pride is at stake and I cannot look the other way”.

### 3.2. Balancing risks and safety

The team is no stronger than its weakest link; every profession has its own perspective, and it is not always easy to decide which perspective should have precedence throughout intraoperative care. Balancing between risks and safety is an essential part of caring for patients and preventing SSIs.

The composition of the team influences how SSI prevention is conducted. When the atmosphere in the OR feels comfortable and safe, the team members feel connected and confident in one another. This comradery and trust creates a sense of work satisfaction and confidence in the professional skills of one’s colleagues. However, a lack of trust in one’s colleagues creates anxiety among the team, a milieu that will not benefit the patient: “You really need your team and it’s important that everybody understands why we do certain things, not just doing it because I say so”.

The team requires a significant amount of hours in the OR to develop a sense of a team. A closely united team increases the pressure to perform at a high level. Another important part of balancing risks and safety is to offer stable platforms where the team can come together and develop preventive work in a constructive and evidence-based way. A well-functioning relationship between team members, both separately and together, helps the team evaluate their own as well as their colleagues’ strengths and shortcomings, knowledge that each member can use to adapt his or her own behavior. As the team sometimes faces difficult adjustments and decisions during surgery, the team members’ mutual confidence could influence how and if decisions are made. A confident team has the ability to smoothly balance unexpected situations:

The surgeons will rather work with an operating room nurse who they know and who are familiar with the surgical procedure. That’s how it is: you work better with some people and not so well with others.

Friction among team members is evident when one or several team members are unwilling to understand other professionals’ responsibilities and competencies. A dysfunctional team can threaten patient safety. Confronting colleagues irrespective of their position requires a security in one’s own competence and a security in the team’s willingness to hear potentially uncomfortable feedback, competencies that develop with experience:

Some people are more or less frightened of some surgeons and then you become nervous and that leads to insecurity and mistakes. For example, if a surgeon is intimidating, I might make mistakes, get nervous and take the surgical towel that I had for cleaning instruments and put it in an open wound during hip replacement.

### 3.3. Legitimacy improves stability in a complex milieu

Prevention of SSIs demands responsibility from each individual in all levels of the organization. Genuine leadership offers the space and conditions to develop prevention in a sustainable and long-term perspective. Professionals, irrespective of affiliation, provide opportunities for improving patient care, the ultimate goal of the profession. Effective leadership helps team members develop confidence in organizational structures and offers stability for the team members.

Managing both team collaboration and organisation are intertwined and clearly related to intraoperative prevention of SSIs. Traditionally, the head surgeon is the team leader and this person’s effectiveness as a leader ensures the effectiveness of the preventive work. Both formal and informal leaders dictate the terms of the preventive work; if they aim for the same goal, it is possible to reach mutual strategies.

The traditional hierarchy, often a safe frame of work organisation, can imbue the team with a sense of stability. This direct and clear resolution feels comfortable, especially in stressful situations that arise from unexpected surgical situations. However, this traditional hierarchy can sometimes threaten openness and ability to function as a team. OR nurses often feel their contributions are minimised. The balance between the legitimacy of OR nurses and the authority of the traditional hierarchy, which places surgeons at the top, is fragile. Prevention of SSIs often end up being a secondary priority, a lack of commitment that often leaves OR nurses feeling ignored:

You can get so tired of yourself and you feel like a disc that repeats itself over and over again, but you can’t give up and capitulate to what you believe is correct. Who will take an interest in SSI prevention if not me, no one would care about that.

Creating all-embracing importance of an optimal surgical environment builds OR nurses’ confidence to function as a key professional for prevention of SSIs:

We need more OR nurses; it’s a little bit scary what I read in the newspaper is that we can be replaced by people without our competence. I can see big risks in SSI prevention if someone without my education suddenly should do my job.

The legitimacy of OR nurses’ competency does not come suddenly, with a specific title or experience; rather, legitimacy is earned by a person who provides assiduous effort towards a goal that is important and obvious to the team. A professional’s legitimacy is determined by others after being evaluated for knowledge related to professional skills. The legitimacy of OR nurses resides in their responsibility to ensure that team members follow hygienic guidelines inside the OR. Creating legitimacy in the complex milieu of an OR requires balancing between one’s own professional perspective and other team members’ perspectives of what is best practice for the patient. In the end, humility is a key attribute for establishing professional legitimacy in the complex milieu of an OR.

## 4. Discussion

The results reveal that the prevention of SSI depends not only on the function of the team but also on organisational and individual legitimacy. In addition, organisational and individual legitimacy depend on each other in a complex caring environment such as the OR.

The OR nurse is responsible for maintaining an optimal hygienic environment inside the OR to ensure intraoperative prevention of SSIs. However, OR nurses experience frustration and sometimes resignation concerning this responsibility. To ensure an optimal environment in the OR demands that the whole team be committed to the prevention of SSIs. As different team members may have different priorities than maintaining proper SSIs prevention procedures, OR nurses can experience feelings of not being heard or taken seriously. These differences in perspective according to professional background reflect the difficulties associated with collaboration. A recently published study shows that nurses on surgical teams often have difficulties speaking up, expressing disagreement, and discussing errors (Kuy & Romero, 2017). Often, these issues include gender or traditional hierarchy issues. Furthermore, 30–40% of OR nurses feel mentally stressed pre- and intraoperatively (Sonoda, Onozuka, & Hagihara, 2018). Negative stress affects surgical performance in the form of ineffective communication and judgment (Arora et al., 2010). The present study shows that experienced OR nurses who have obtained legitimacy from other team members feel more prepared than their inexperienced colleagues to deal with difficult situations that appear during a surgical procedure. Clearly, OR nurses need more support when it comes to expressing their concerns within the surgical team milieu (Sonoda et al., 2018). That is, a prolonged investment in creating a supportive working environment will help OR nurses improve the prevention of SSIs and ultimately the care provided to the patient.

Prevention of SSI is an essential part of the OR nurse’s responsibilities, but evidence from many care bundles is not clear and creates doubts. A British study shows that physicians have a tendency to reject written guidelines and instead adhere to acceptable behaviour defined by the medical profession; however, the same study found that nurses tend to see following written guidelines as synonymous with professionalism (McDonald, Waring, Harrison, Walshe, & Boaden, 2005). This perspective makes it understandable how difficult it is to develop a cohesive unit. In this study, OR nurses experience that other team members do not prioritise complying with SSI prevention measures, an attitude that makes creating an optimal surgical environment difficult. As in this study, another study points out that there remains an underestimated effect on implementing care bundles (Koek et al., 2017). There is a need for awareness of the impact on specific competencies on patient outcome (Meretoja & Koponen, 2012), and this is obvious when it comes to prevention of SSIs. There is a logical connection between education, clinical judgment, and patient outcomes (Gillespie, Harbeck, Falk-Brynhildsen, Nilsson, & Jaensson, 2018).

The connection between the function of the team and the risk of SSIs is an important finding that needs to be addressed in the management of OR nursing. The amount of time it takes to create a well-functioning team is significant. The complexity of modern surgery demands teams that are skilled, safe, and confident in their individual roles. Teams that stay cohesive for long periods have opportunities to develop their collaboration, which might improve patient outcomes in general (Özdemir-van Brunschot et al., 2015). This understanding means that conflicts within teams might compromise patient safety, including the prevention of SSI. Therefore, management, the ultimate authority for the organization, must find ways to address conflicts, which might also include ways to encourage open and trusting team work. That is, as collaboration is important for all teams, management should train teams in collaborative team work strategies (Kuy & Romero, 2017). Team members who work together but with their own goals are ineffective (Cvetic, 2011). These sorts of teams increase the risk of SSIs as these teams do not have confidence and respect for different occupational skills. Using the expertise of OR nurses is a strong predictor for SSIs prevention (Bathish, McLaughlin, & Talsma, 2015). As surgical methods have developed rapidly, OR nurses have been required to constantly develop and extend their specific competencies (Meretoja & Koponen, 2012). The results in our study suggest that cohesive teams with specific competencies and long-term collaboration may help ensure that SSIs prevention measures are used. For example, a US validated survey of perioperative safety showed that improving all dimensions of teamwork improves patient outcomes (Molina et al., 2016). Teams with long-term experience of working together can combine their skills and plan for safe perioperative care (Silén‐Lipponen, Tossavainen, Turunen, & Smith, 2005). Moreover, a high-quality interdisciplinary team is characterised by collegial support and openness (Rydenfält, Johansson, Larsson, Åkerman, & Odenrick, 2012).

Organizational determinants have a great impact on professionals when it comes to providing conditions for perioperative care and ensuring patient safety (Sevdalis, Hull, & Birnbach, 2012). Team members often have different department heads and that results in difficulties finding platforms where they can develop and implement mutual goals. Therefore, management should develop organizational structures for the team to come together as a cohesive unit. To ensure the sustained presence of OR nurses’ competencies during intraoperative care, there is a need to capitalise on their important contributions to the prevention of SSIs. If the prevention of SSIs becomes a key assignment with legitimacy, OR nursing will remain a lasting and respected profession.

### 4.1. Methodological considerations

In this study, a phenomenological-based qualitative method was used to gather experiences and meanings. The results of this study attest to the fruitfulness of a descriptive method. Trustworthiness has been an important factor for the authors, who strive to take actions to ensure the bridling of the preunderstanding. Throughout the research process, the authors openly questioned each other’s assumptions. By being open, curious, and reflexive towards the phenomenon during both the interviews and the analysis, the authors ensured the credibility of the results. When searching for meanings of a phenomenon, the challenge is to avoid forming conclusions too quickly and therefore drawing conclusions using insufficient data (Dahlberg & Dahlberg, 2019). During the interviews, the interviewer avoided using leading questions by allowing the participants to freely talk about their experiences. During the interview, follow-up questions were asked: “Could you give me an example?”; “How do you mean?”; and “Can you describe in more detail?”. The interviews were thus characterised by openness and flexibility. As the OR context is very specific and results only come from OR nurses’ perspectives, transferability to other contexts is hard to ascertain. However, this study was carried out in seven hospitals, which makes it possible to apply findings to other ORs.

## 5. Conclusion

By setting up mutual platforms and forums for quality development, increasing legitimacy for OR nurses and establishing fixed teams, prevention of SSIs will continue to improve, ensuring the patients’ safety during intraoperative care. Prevention becomes more and more urgent as antibiotic resistance increases. We can no longer accept avoidable risks such as SSIs. Reducing bacterial load inside OR should be a high priority for all surgical settings. Traditional hierarchy inside an OR also needs to be challenged for the possibility of moving modern surgery into the future.
